# Linking women’s empowerment to modern contraceptive use: evidence from the Tanzania demographic and health survey 2022

**DOI:** 10.1186/s12905-025-04153-9

**Published:** 2025-11-29

**Authors:** Mihee Kim, Heeran Chun, Sung-il Cho, Minah Kang

**Affiliations:** 1 Department of Public Health Science, Graduate School of Public Health, Seoul National University, 1, Gwanak-ro, Gwanak-gu, Seoul, 08826 Republic of Korea; 2 Department of Health and Welfare, Jungwon University, 85, Munmu-ro, Goesan-eup, Goesan-gun, Chungcheongbuk-do 28024 Republic of Korea; 3 Department of Public Administration, Ewha Womans University, 52, Ewhayeodae-gil, Seodaemun-gu, Seoul, 03760 Republic of Korea

**Keywords:** Contraception, Women’s empowerment, Family planning, Modern contraceptive use, Reproductive health, Sustainable development, Gender equality

## Abstract

**Background:**

High fertility rates in sub-Saharan Africa underscore the importance of contraceptive use in managing population growth. While previous studies have linked women’s empowerment to contraceptive behavior, this study adopts a multidimensional approach, emphasizing decision-making autonomy, resource access, and negotiation skills. By examining these distinct dimensions, the study provides a more comprehensive understanding of how empowerment influences modern contraceptive use among women in Tanzania, offering insights for targeted reproductive health strategies.

**Methods:**

This cross-sectional study analyzed data from the 2022 Tanzania Demographic and Health Survey (TDHS), which focuses on 8236 currently married, non-pregnant women aged 15–49 years. The empowerment indicators included participation in household decision-making, attitudes toward intimate partner violence, negotiation of sexual relationships, property ownership, and social independence. Descriptive and bivariate analyses were conducted, followed by multivariate logistic regression to assess associations. The analyses incorporated complex survey design adjustments for national representativeness.

**Results:**

Modern contraceptive use was reported by 34.9% of women. The significant factors associated with greater contraceptive use included education, household wealth, employment, media exposure to family planning messages, and empowerment indicators. Women participating in all three household decision-making domains (AOR 1.64, 95% CI 1.36–1.98), negotiating sexual relationships (AOR 1.41, 95% CI 1.19–1.67), and owning both a house and land (AOR 1.47, 95% CI 1.27–1.70) presented significantly higher odds of modern contraceptive use.

**Conclusions:**

Women’s empowerment significantly influences modern contraceptive use. Enhancing women’s autonomy, decision-making power, and socioeconomic opportunities should be prioritized in family planning initiatives. Addressing structural and social barriers that limit women’s agency will help ensure informed reproductive health choices, ultimately promoting gender equity and sustainable development.

## Introduction

Tanzania has experienced substantial population growth over the past five decades, primarily driven by persistently high fertility rates. Since the initial census in 1967, which recorded a population of 12.3 million, the country’s population has grown nearly fivefold, reaching 61.7 million in 2022 [1]. By 2030, this figure is projected to rise further to approximately 87 million [2]. This growth can be attributed mainly to sustained high fertility, declining mortality, and to a lesser extent, cross-border migration from neighboring countries [3, 4]. Among these factors, high fertility remains the dominant contributor [5]. The 2022 Tanzania Demographic and Health Survey (TDHS) reported a modest decline in the total fertility rate (TFR) from 5.2 in 2015/16 to 4.8 in 2022 [6]. Despite this reduction, Tanzania’s TFR remains significantly higher than those of neighboring sub-Saharan African countries, which have successfully lowered fertility rates through effective, high-quality family planning programs. For example, Kenya reported a TFR of 3.42, Rwanda 3.99, and Malawi 4.13 [7].

The decline in fertility rates is closely associated with contraceptive use, a relationship extensively examined in the literature. Early theoretical frameworks, such as those proposed by Bongaarts [8], highlighted contraception as a deliberate means of regulating marital fertility and emphasized its critical role in fertility reduction. Subsequent empirical studies have reinforced this viewpoint. Cleland et al. [9], for example, reported that increased contraceptive use accounted for approximately 75% of the fertility decline observed in developing countries over the past six decades. Regional data support these findings. Kenya, Rwanda, and Malawi, all of which have actively promoted voluntary and equitable access to family planning in recent decades, have attained high modern contraceptive prevalence rates (mCPR): 57.0% in Kenya [10], 58.4% in Rwanda [11], and 65.0% in Malawi [12]. In contrast, Tanzania’s mCPR remains relatively low at 31.0% [6], though this rate is slightly above the sub-Saharan African average of 22.0% [13].

In addition to contraceptive use, women’s empowerment is widely acknowledged as a fundamental determinant of fertility outcomes. A systematic review underscored the transformative impact of empowerment on reproductive health, linking higher levels of empowerment to lower fertility rates, fewer unintended pregnancies, and increased birth intervals [14]. Similarly, a comprehensive review identified contraceptive use as the most commonly studied outcome in family planning research and consistently found a positive correlation with women’s empowerment [15]. Building on this foundation, evidence from low- and middle-income countries demonstrates that women’s empowerment is positively associated with modern contraceptive use. In sub-Saharan Africa, studies from Nigeria, Niger, and Ethiopia highlight the role of household decision-making and gender-equitable attitudes in promoting contraceptive uptake [16–18]. Similar patterns have been observed in South and Southeast Asia, including Bangladesh and Pakistan, where women’s decision-making authority and inclusive family planning approaches are linked to higher contraceptive use [19, 20]. In Tanzania, both regional and national data confirm this relationship [21, 22]. Multi-country analyses across sub-Saharan and East Africa further affirm the consistent effect of empowerment on contraceptive adoption [23, 24].

Empowerment is conceptually understood as a multidimensional process. Kabeer [25] defines empowerment as the “process by which those who have been denied the ability to make strategic life choices acquire such an ability.” This framework outlines three interrelated dimensions of empowerment: agency, resources, and achievements [26]. Furthermore, empowerment is broadly conceptualized as the “expansion of freedom of choice and action to shape one’s life” [27], emphasizing both the transformative process and individual agency. This process enhances women’s decision-making capabilities, whereas the agency facilitates their meaningful involvement in decision-making activities [28]. In the context of reproductive health, empowerment is essential for enabling women to make informed choices and take intentional action, especially regarding modern contraceptive use [29]. Empowerment enhances women’s autonomy, enabling them to exercise greater control over their reproductive health and overall well-being [30].

Recent thinking extends this understanding to include not only the choice to use contraception but also the choice not to—provided the decision is voluntary, informed, and free from coercion. Holt et al. [31] describe this as contraceptive agency, or the capacity to decide whether or not to prevent or delay pregnancy and to act on that decision independently. Empirical evidence supports this perspective. A qualitative study in Mwanza, Tanzania, found that women often exercised autonomy within complex social environments where external influences shaped both their use and non-use of contraception [32]. Likewise, a quantitative study among adolescent and young adult females in Uganda showed that many contraceptive decisions were not made individually, further highlighting the need to recognize voluntary non-use as a potential indicator of reproductive agency [33].

Despite its acknowledged significance, women’s empowerment remains a contextual and multidimensional construct [15], with ongoing debates regarding the appropriate indicators for its measurement. Efforts to conceptualize women’s empowerment have extended beyond African contexts to include global perspectives. For example, the Survey-based Women’s Empowerment Index (SWPER), initially designed to measure empowerment within the African context [30], was later expanded into SWPER Global by Ewerling et al. [34]. This index comprises three dimensions: “attitudes toward violence”, “social independence”, and “decision-making” [34]. In Tanzania, Mganga et al. [35] developed a tailored empowerment index utilizing data from the TDHS (2004/05, 2010, and 2015/16) to evaluate six domains: “attitudes toward violence”, “decision-making”, “social independence”, “access to healthcare”, “age at critical life events”, and “property ownership.”

In particular, a review article on women’s empowerment and family planning identified ‘mobility’ and ‘participation in household decision-making’ as two of the most frequently evaluated indicators of empowerment [15]. These indicators encompass specific domains of decision-making, including access to medical care, the management of significant or minor household expenditures, and decisions regarding visits to relatives or friends. These measures function as proxies for women’s empowerment and are instrumental in assessing family planning outcomes, particularly contraceptive use. Additionally, another review of empowerment measures within family planning programs emphasized the importance of validated indicators, such as gender equality attitudes and decision-making authority, for assessing how empowerment influences family planning outcomes [36].

Despite growing research interest, many studies conducted in Tanzania have relied on limited conceptualizations of women’s empowerment, often using binary indicators that overlook its multidimensional nature. For example, Nanda et al. [37] found that contraceptive use was significantly associated with wives’, but not husbands’, equitable gender attitudes, highlighting the influence of spousal gender dynamics. A qualitative study by Schuler et al. [38] reported that 91% of non-users desired to space or limit births but were deterred by prevailing gender norms and misinformation. Using TDHS 2010 data, Kidayi et al. [22] showed that empowered women were 1.4 times more likely to use modern contraception (OR = 1.40; 95% CI: 1.13–1.63), although empowerment was treated as a binary construct, limiting interpretability. More recent research has identified additional relevant factors. Mwalupani et al. [39] emphasized the role of economic resources in informed decision-making in contraceptive practices, while Yussuf et al. [40] highlighted the influence of spousal agreement on desired family size. Although these studies affirm the relevance of empowerment, few have adopted multidimensional frameworks to explore how specific empowerment domains relate to contraceptive behavior. To address this gap, the present study employs a broader analytical approach that captures the complexity of empowerment and evaluates its distinct dimensions in the Tanzanian context.

Given the multidimensional and context-specific nature of women’s empowerment, it is essential to incorporate country-specific factors when designing family planning policies and interventions. As part of the Family Planning 2030 commitments, Tanzania has set a target to increase its mCPR from 31% in 2022 to 42% by 2025 [41]. However, progress has been slow, with annual growth in mCPR averaging only 1%, as shown by trends from 27% in 2010 to 32% in 2015/16 and 31% in 2022 [6]. To accelerate progress, targeted and evidence-based interventions must be implemented, tailored to Tanzania’s unique sociocultural context, and grounded in a comprehensive understanding of women’s empowerment.

Women’s empowerment is also central to global development agendas. Sustainable Development Goal (SDG) 5 recognizes empowerment as a foundation for advancing reproductive health and family planning. Empowerment also plays a vital role in reducing gender inequality [42]. Tanzania’s commitment to achieving the SDGs highlights the importance of expanding access to contraception and implementing effective family planning policies to increase modern contraceptive use, thereby contributing to broader goals of global health and gender equality [43].

Therefore, this study aims to examine the relationship between women’s empowerment and modern contraceptive use among currently married, non-pregnant women aged 15–49 years in Tanzania, focusing on multiple distinct dimensions of empowerment. The findings are expected to provide valuable evidence for designing targeted family planning policies and interventions tailored to Tanzania’s sociocultural context, while contributing to global efforts to enhance reproductive health and advance the Sustainable Development Goals.

## Methods

### Study setting and sampling

This study employed secondary data from the 2022 TDHS and adopted a cross-sectional research design to explore whether empowerment influences the utilization of modern contraceptives. The TDHS is a nationally representative household survey conducted approximately every five years to obtain indicators related to fertility, family planning, and maternal and child health. The 2022 TDHS was implemented by the National Bureau of Statistics in collaboration with the Office of the Chief Government Statistician of Zanzibar, with technical assistance from ICF and funding from the United States Agency for International Development (USAID). The DHS targeted women aged 15–49 and men aged 15–59 residing in selected households across Tanzania. In total, 16,312 households were selected using a two-stage cluster sampling design, achieving a 97% response rate. Among these households, 15,254 women aged 15–49 were successfully interviewed. Comprehensive details regarding the sampling procedure are provided in the TDHS 2022 final report [6].

On the basis of the research questions, the analysis concentrated on married or cohabiting respondents, as the empowerment-related questions were specifically designed for this group. Pregnant women were excluded to ensure that current contraceptive use reflected active fertility preferences. All variables included in the final regression model had complete data, with no missing values detected; thus, no imputation or casewise deletion was necessary. After applying these criteria, the final weighted analytic sample comprised 8236 currently married, non-pregnant women of reproductive age.

### Measurements

#### Outcome variables

The outcome variable, defined on the basis of Hubacher and Trussell’s [44] description, refers to modern contraceptive methods as “a product or medical procedure that interferes with reproduction from acts of sexual intercourse.” Modern contraceptive use was assessed via responses to two questions from the TDHS women’s questionnaire: (1) “Are you or your partner currently doing something or using any methods to delay or avoid pregnancy?” and (2) “Which method are you using?” In the TDHS 2022, current contraceptive methods were classified into three groups: ‘No Method,’ ‘Traditional Method,’ and ‘Modern Method.’ Modern methods included female and male condoms, female and male sterilization, implants, intrauterine devices (IUDs), injectables, oral contraceptives (pill), emergency contraception, the standard days method (SDM), and the lactational amenorrhea method (LAM). Traditional methods included periodic abstinence (calendar or rhythm methods), withdrawal, and other traditional approaches. For this study, contraceptive use was ultimately recoded as a binary variable, with ‘Yes’ indicating the use of modern methods and ‘No’ representing the use of either no method or traditional methods.

#### Independent variables

The demographic characteristics of the women included variables related to age at critical life events, residence, fertility, education, wealth, employment, and exposure to family planning information. Women’s current age was grouped into four age groups: 15–19, 20–29, 30–39, and 40–49 years. Age at first cohabitation or marriage was classified into three categories on the basis of quantiles: under 18, 18–20, and over 21 years. Fertility was measured using the number of surviving children and divided into tertiles: 2 or fewer, 3–4, and 5 or more. Residence was recorded as either rural or urban. Education was categorized into four groups: no education, primary, secondary, and higher. Household wealth was measured via the wealth index, which classifies households into five quintiles: poorest, poor, middle, richer, and richest. The wealth index, derived through principal component analysis, considers household characteristics and asset ownership [45]. Employment for payment was assessed on the basis of whether respondents earned income from employment within the past 12 months. Women were classified as ‘employed for cash’ if they received payments either in cash only or in both cash and in kind. Women who were employed and received payments in kind only or were unpaid were classified as ‘employed not for cash’. Exposure to family planning messages was divided into two groups, ‘yes’ or ‘no’, on the basis of whether respondents had obtained family planning information from any of the listed sources during the previous 12 months: radio, television, newspapers or magazines, mobile phone messages (via voice or text), social media platforms (e.g., Facebook, Twitter, or Instagram), posters, leaflets, brochures, outdoor signs or billboards, and community meetings or events.

#### Women’s empowerment variables

The primary independent variable was women’s empowerment, measured using indicators derived from indices developed to assess empowerment in African contexts [30, 46], across low- and middle-income countries [34], and with specific relevance to Tanzania [35, 47]. Relevant variables from the TDHS were selected based on prior evidence, covering domains such as decision-making, attitudes toward violence, social independence, and property ownership.

Decision-making was assessed based on women’s involvement in three key household decisions: (1) their own health care, (2) major household purchases, and (3) visits to family or relatives. Each domain was coded as a binary variable, with ‘Yes’ indicating participation—either independently or jointly with a partner—and ‘No’ indicating no involvement. A composite variable was then constructed to reflect the level of participation, categorized into three graded groups: ‘All three decisions,’ ‘1–2 decisions,’ and ‘None.’

Attitudes toward violence were measured on the basis of responses to five scenarios where wife-beating might be considered justified: (1) if the wife goes out without informing her husband, (2) neglects the child, (3) argues with her husband, (4) refuses to have sex relations with her husband, and (5) burns food. Each scenario was recoded as a binary variable, with ‘Yes’ indicating that the respondent considered violence justified and ‘No’ indicating that it was either unjustified or the respondent was unsure. A summative score ranging from 0 to 5 was then calculated to reflect the number of scenarios where violence was justified. This score was subsequently recoded into a binary variable: ‘Yes’ if violence was justified in at least one scenario, and ‘No’ if it was not justified in any.

Social independence was measured using six binary indicators informed by prior research [35, 36], encompassing ownership and use of financial tools and media exposure. Specifically, three indicators—ownership of a mobile phone, ownership of a bank account, and use of a mobile phone for financial transactions—were coded as ‘Yes’ or ‘No’ based on classifications in the original TDHS dataset. Media exposure was assessed through three items indicating whether the respondent engaged with newspapers or magazines, radio, or television at least once a week or less; any exposure was coded as ‘Yes,’ and no exposure as ‘No.’ Each ‘Yes’ response contributed one point to a composite score ranging from 0 to 6. This score was then categorized into three levels of social independence: ‘Low’ (0–1 points), ‘Moderate’ (2–3 points), and ‘High’ (4–6 points).

Property ownership was determined by whether women owned a house or land, either individually or jointly. Ownership—whether solely, jointly with a spouse or others, or both—was coded as ‘Yes’; lack of ownership was coded as ‘No.’ An aggregate variable was then constructed and categorized into three levels: ‘Both house and land,’ ‘Either house or land,’ and ‘No property ownership.’

Furthermore, previous studies have noted that sexual decision-making is a significant factor influencing family planning and reproductive health outcomes [15, 36]. In line with this, the present study explored women’s gender-equitable attitudes toward sexual and reproductive health by examining their ability to negotiate sexual relationships. Sexual empowerment, in this context, was assessed through two indicators: the ability to reject sexual relations and the ability to advocate for condom use. Initially, a binary variable was developed for each indicator, with responses dichotomized as ‘Yes’ or ‘No.’ Subsequently, a synthesized variable was generated to capture the level of sexual negotiation ability, categorized as follows: ‘Can refuse sex and ask for condom use’, ‘Can refuse sex or ask for condom use’, and ‘Cannot do both’.

### Data analysis

Descriptive statistics summarized the demographic and empowerment variables. Bivariate analyses via Pearson’s chi-square tests were used to examine associations between modern contraceptive utilization and explanatory variables. Multivariate logistic regression was employed to evaluate these associations, adjusting for potential confounders, and the results are presented as adjusted odds ratios (AORs) with 95% confidence intervals (CIs). A threshold of *P* < 0.05 was used to determine statistical significance. Before model fitting, pairwise Pearson correlation coefficients were computed to assess the degree of association between the empowerment domains, and variance inflation factors (VIFs) were used to evaluate multicollinearity among covariates. All analyses considered a complex survey design, incorporating clustering, stratification, and sampling weights, and were conducted via the “Survey” package in R (version 4.4.1).

## Results

### Descriptive characteristics

Table 1 indicates that 34.9% of currently married women in Tanzania reported using modern contraceptives. The mean age was 32.5 years, and the mean age at first marriage or cohabitation was 19.5 years. Over two-thirds (73.5%) of the women had fewer than five living children. The majority of women (68.1%) resided in rural areas. Only 21.1% of women had attained secondary or higher education, and 42.2% were employed for cash. Most women (80%) reported exposure to family planning information.

**Table 1:** Demographic characteristics and modern contraceptive use distribution among currently married, non-pregnant women, Tanzania DHS 2022

Demographic characteristics		Women		Modern contraceptive use	*P*-value
		*N*	%	%	
Total		8236	100.0	34.9	
Current age					
	Mean ± SD	32.47 ± 0.14			
	15–19 years	431	5.2	19.9	<0.001
	20–29 years	3019	36.7	37.8	
	30–39 years	2683	32.6	39.6	
	40–49 years	2102	25.5	27.9	
Age at first cohabitation					
	Mean ± SD	19.45 ± 0.08			
	Under18 years	3130	38.0	33.3	0.051
	18–20 years	2559	31.1	37.2	
	Over 21 years	2547	30.9	34.7	
Number of living children					
	Mean ± SD	3.36 ± 0.04			
	Less than 2	3365	40.9	32.0	<0.001
	3–4 Children	2682	32.6	40.4	
	5 or more	2189	26.6	32.8	
Residence					
	Urban	2624	31.9	38.7	0.002
	Rural	5612	68.1	33.2	
Education level					
	No education	1686	20.5	25.1	<0.001
	Primary	4814	58.5	36.9	
	Secondary	1636	19.9	39.3	
	Higher	99	1.2	34.7	
Wealth index					
	Poorest	1482	18.0	23.6	<0.001
	Poorer	1505	18.3	32.0	
	Middle	1595	19.4	38.8	
	Richer	1775	21.6	40.7	
	Richest	1878	22.8	37.5	
Employment					
	Not employed	2556	31.0	29.9	<0.001
	Employed for cash	3476	42.2	39.3	
	Employed not for cash	2204	26.8	33.9	
Heard/seen about family planning					
	Yes	6588	80.0	37.1	<0.001
	No	1648	20.0	26.2	

Bivariate analysis results in Table 1 show significant associations between modern contraceptive use and all demographic variables, except age at first cohabitation. The uptake of modern methods was highest among women aged 30–39 years (39.6%), those with 3–4 children (40.4%), urban residents (38.7%), women with secondary education (39.3%), those employed for cash (39.3%), women from wealthier households (40.7%), and women who heard or saw family planning messages via mass media (37.1%).

### Distribution of women’s empowerment in Tanzania

Table 2 indicates that the majority of respondents participated in household decision-making, with 74.3% involved in decisions about personal healthcare, 64.7% in major household purchases, and 68.6% in visiting families or relatives. Overall, 54.9% of women participated in all three decisions, 27.8% in one or two decisions, and 17.3% did not participate in any household decisions. Regarding attitudes toward violence, 48.0% of women indicated that violence was not justified in any situation, whereas 52.0% believed it was justified in one or more scenarios. Specifically, 34.8% justified violence if they went out without informing their husbands, 41.2% if they neglected their children, 37.7% if they argued with their husbands, 28.5% if they refused sexual relations, and 13.8% if they burned food. In terms of sexual decision-making, 68.7% of the women reported the ability to reject sexual relations, and 58.6% could ask for condom use. Overall, 51.3% had both abilities, whereas 24.0% lacked both abilities. For asset ownership, most women (62.2%) did not own land, and 46.1% reported not owning a house. Among the respondents, 34.5% owned both land and a house, 22.7% owned either land or a house, and 42.9% owned neither. In the social independence domain, 61.6% of women owned a mobile phone, but only 6.8% had a bank account. Among mobile phone users, 44.3% utilized their phones for financial transactions. Media exposure was reported by 19.9% of women who read newspapers, 55.8% who listened to the radio, and 42.8% who watched television regularly. Overall, 28.2% of women scored high (4–6 points) in the social independence domain, whereas 36.8% were classified as having low social independence (0–1 point).

**Table 2:** Women’s empowerment and modern contraceptive use distribution among currently married, non-pregnant women, Tanzania DHS 2022

Women’s empowerment variables			Women		Modern contraceptive use	*P*-value
			*N*	%	%	
Decision-making in household						
	Decision maker on own health care					
		Yes = Respondent alone, Respondent and husband/partner	6122	74.3	37.8	<0.001
		No = Husband/partner alone, Someone else, Other	2113	25.7	26.7	
	Decision maker on major household purchases					
		Yes = Respondent alone, Respondent and husband/partner	5330	64.7	38.0	<0.001
		No = Husband/partner alone, Someone else, Other	2906	35.3	29.4	
	Decision maker on visits to family or relatives					
		Yes = Respondent alone, Respondent and husband/partner	5646	68.6	37.4	<0.001
		No = Husband/partner alone, Someone else, Other	2589	31.4	29.5	
	Participation in decision-making					
		All 3 decisions	4525	54.9	38.0	<0.001
		1–2 decisions	2569	27.8	36.3	
		None	1420	17.3	23.0	
Attitude towards violence						
	Justified if wife goes out without telling husband					
		No	5370	65.2	35.6	0.203
		Yes	2865	34.8	33.7	
	Justified if wife neglects the children					
		No	4839	58.8	35.1	0.744
		Yes	3396	41.2	34.7	
	Justified if wife argues with husband					
		No	5132	62.3	36.3	0.007
		Yes	3104	37.7	32.6	
	Justified if wife refuses to have sex with husband					
		No	5885	71.5	35.4	0.310
		Yes	2350	28.5	33.8	
	Justified if wife burns the food					
		No	7098	86.2	35.4	0.153
		Yes	1138	13.8	32.1	
	Justified for any of the five reasons					
		No	3949	48.0	35.6	0.362
		Yes	4286	52.1	34.4	
Sexual empowerment						
	Can refuse sex					
		Yes	5654	68.7	37.8	<0.001
		No	2581	31.3	28.6	
	Can ask partner to use a condom					
		Yes	4826	58.6	39.1	<0.001
		No	3410	41.4	29.0	
	Ability to negotiate sexual relations					
		Can refuse sex and ask for condom use	4223	51.3	39.4	<0.001
		Can refuse sex or ask for condom use	2295	24.8	34.1	
		Cannot do both	1978	24.0	26.2	
Property ownership						
	Owns a house alone or jointly					
		Yes = Alone only, Jointly only, Both alone and jointly	4436	53.9	36.3	0.045
		No = Does not own	3800	46.1	33.4	
	Owns land alone or jointly					
		Yes = Alone only, Jointly only, Both alone and jointly	3110	37.8	38.4	<0.001
		No = Does not own	5126	62.2	32.8	
	Ownership of assets					
		Both house and land	2842	34.5	38.5	0.001
		Either of house or land	2096	22.7	33.0	
		No property ownership	3532	42.9	33.1	
Social independence						
	Owns a mobile phone					
		Yes	5072	61.6	37.7	<0.001
		No	3163	38.4	30.5	
	Owns a bank/financial account					
		Yes	562	6.8	37.8	0.267
		No	7674	93.2	34.7	
	Uses mobile phone for financial transactions					
		Yes	3644	44.3	39.6	<0.001
		No	4592	55.8	31.3	
	Frequency of reading newspaper or magazine					
		Yes = At least once a week, Less than once a week	1636	19.9	35.7	0.557
		No = Not at all	6599	80.1	34.7	
	Frequency of listening to radio					
		Yes = At least once a week, Less than once a week	4592	55.8	38.0	<0.001
		No = Not at all	3644	44.2	31.0	
	Frequency of watching television					
		Yes = At least once a week, Less than once a week	3523	42.8	38.3	<0.001
		No = Not at all	4713	57.2	32.4	
	Social independence					
		High (4–6 points)	2325	28.2	38.4	<0.001
		Moderate (2–3 points)	2880	35.0	38.4	
		Low (0–1 points)	3030	36.8	29.0	

Modern contraceptive use was significantly associated with multiple dimensions of women’s empowerment, including household decision-making, sexual autonomy, property ownership, and social independence. Modern method uptake was higher among women who were involved in all three domestic decision-making areas (38.0%), those who rejected violence in situations involving arguments with their husbands (36.3%), and those who reported both the ability to refuse sexual relations and to request condom use (39.4%). Higher contraceptive use was also observed among women who owned both land and a house (38.5%) and those classified as having high social independence (4–6 points) (38.4%).

### Association between women’s empowerment and modern contraceptive use

Table 3 illustrates the results of the survey-weighted logistic regression analysis. Household decision-making proved to be a crucial factor. Women who participated in all three decision areas had 1.64 times greater odds of using modern methods (95% CI: 1.36 − 1.98), and those involved in one or two decisions had 1.54 times higher odds (95% CI: 1.27–1.88) than those with no engagement. Sexual empowerment also played a substantial role. Greater odds of modern contraceptive use were observed among women who could both refuse sex and request condom use (AOR = 1.41, 95% CI: 1.19–1.67), followed by those with either ability (AOR = 1.21, 95% CI: 1.01–1.45), relative to those lacking both. Property ownership also matters. Owning both a house and land was associated with a 47% increase in the likelihood of using modern contraceptives (95% CI: 1.27–1.70) relative to women with no assets. However, the composite measure of social independence was not significantly associated with contraceptive use. Similarly, attitudes toward violence were not a significant factor.

**Table 3:** Logistic regression on contraceptive use by women’s empowerment and demographic variables (modern vs. traditional or none)

Variables		AOR	95% CI		*P*-value
			Lower	Upper	
Participation in decision-making					
	All 3 decisions	1.64	1.36	1.98	<0.001
	1–2 decisions	1.54	1.27	1.88	<0.001
	None	1.00			
Attitude towards violence					
Justified for any of the five reasons					
	No	1.02	0.91	1.15	0.746
	Yes	1.00			
Ability to negotiate sexual relations					
	Can refuse sex and ask for condom use	1.41	1.19	1.67	<0.001
	Can refuse sex or ask for condom use	1.21	1.01	1.45	0.040
	Cannot do both	1.00			
Property ownership					
	Both house and land	1.47	1.27	1.70	<0.001
	Either of house or land	1.01	0.85	1.19	0.947
	No property ownership	1.00			
Social independence					
	High (4–6 points)	0.93	0.74	1.17	0.548
	Moderate (2–3 points)	1.07	0.91	1.26	0.408
	Low (0–1 points)	1.00			
Current age					
	15–19 years	1.00			
	20–29 years	1.73	1.18	2.53	0.005
	30–39 years	1.47	0.98	2.22	0.065
	40–49 years	0.76	0.50	1.15	0.195
Age at first cohabitation					
	Under18 years	1.00			
	18–20 years	1.07	0.93	1.22	0.363
	Over 21 years	0.94	0.81	1.09	0.393
Number of living children					
	Less than 2	1.00			
	3–4 Children	1.51	1.31	1.75	<0.001
	5 or more	1.47	1.22	1.76	<0.001
Residence					
	Urban	1.00			
	Rural	1.15	0.95	1.39	0.145
Education level					
	No education	1.00			
	Primary	1.36	1.13	1.64	0.001
	Secondary	1.34	1.05	1.73	0.021
	Higher	1.08	0.61	1.90	0.788
Wealth index					
	Poorest	1.00			
	Poorer	1.35	1.08	1.68	0.009
	Middle	1.71	1.36	2.14	<0.001
	Richer	1.86	1.41	2.44	<0.001
	Richest	1.57	1.15	2.14	0.004
Employment					
	Not employed	1.00			
	Employed for cash	1.33	1.15	1.54	<0.001
	Employed not for cash	1.22	1.01	1.47	0.036
Heard/seen about family planning					
	Yes	1.24	1.06	1.45	0.008
	No	1.00			

Regarding demographic factors, women aged 20–29 had 1.73 times higher odds (95% CI: 1.18–2.53) compared to women aged 15–19. Women with 3–4 children had a 51% higher likelihood of using modern contraceptives (95% CI: 1.31–1.75), and those with five or more children had a 47% increase in odds (95% CI: 1.22–1.76), compared to women with fewer than two children. Education showed a positive association. Modern methods were significantly more prevalent among women with primary (AOR = 1.36, 95% CI: 1.13–1.64) and secondary education (AOR = 1.34, 95% CI: 1.05–1.73), relative to those lacking formal education. Women from the richest households had 1.86 times greater odds (95% CI: 1.41–2.44) of using modern contraception than those from the poorest households. Employment status also mattered. Compared to non-employed women, those earning cash income were 33% more likely to use modern contraceptives (95% CI: 1.15–1.54), while women engaged in unpaid work showed a 22% increased likelihood (95% CI: 1.01–1.47). Hearing or seeing family planning messages via mass media was linked with 1.24 times greater odds of contraceptive use (95% CI: 1.06–1.45). Finally, all VIF values were under 1.4, confirming the absence of multicollinearity.

## Discussion

The principal findings of this study show that approximately one-third of currently married, non-pregnant women in Tanzania utilized modern contraceptive methods. Key empowerment variables significantly associated with modern contraceptive use included engagement in household decisions, the ability to negotiate sexual matters, and property ownership, whereas attitudes toward wife beating did not exhibit a significant relationship. In addition, sociodemographic factors such as age, number of surviving children, education, wealth, employment status, and exposure to family planning information were significant predictors of modern methods.

This study found that 34.9% of currently married, non-pregnant women in Tanzania were using modern contraceptives. Although this figure is slightly higher than the national average of 31.0% reported in the 2022 TDHS [6], it remains modest compared to several regional counterparts. Ethiopia and Uganda have reported mCPRs of 40.5% [48] and 39.0% [49], respectively. Malawi stands out with a rate of 65.0% [12], reflecting sustained investments in family planning and broad-based community engagement. In contrast, Nigeria’s mCPR remains low at 12.0% [50], illustrating the wide variation in outcomes across sub-Saharan Africa. A pooled analysis of the region estimated an average mCPR of 22.0% (95% CI: 21.8–22.2%), with country-level rates ranging from 49.7% in Namibia to just 3.5% in the Central African Republic [13]. In this context, Tanzania’s rate sits above the regional average but falls behind high-performing countries, indicating the need for strategic programmatic shifts.

Among empowerment-related factors, a strong association was observed between women’s decision-making authority at home and their use of modern contraception, reflecting the essential impact of empowerment on reproductive health behaviors. This finding aligns with prior studies using comparable indicators. For example, in Nigeria, women who jointly or independently made decisions about large household purchases, family visits, or their healthcare were 1.15–1.60 times more likely to utilize modern contraceptives [16]. In Niger, decision-making involvement was linked to a 1.36-fold increase in contraceptive use, even after adjusting for demographic factors [17]. Similarly, evidence from Egypt showed that women involved in joint or independent decisions were more likely to adopt both short-acting and long-acting contraceptives [51]. These findings collectively suggest that domestic decision-making power is a crucial dimension of women’s agency.

Women’s ability to negotiate sexual matters was positively linked to higher modern contraceptive utilization, further reinforcing the role of personal agency in reproductive choices. In Vietnam, women demonstrating negotiation capacity in sexual and condom-related decisions had 1.60 times higher odds of condom use during their last intercourse and 1.56 times higher odds of consistent condom use in the past 12 months [52]. In Ghana, each one-point increase in a sexual empowerment index was linked to a 24% increase in the odds of current contraceptive use [53]. Consistent with these findings, this study confirms that women’s ability to negotiate sexual matters contributes to higher modern contraceptive use, reinforcing the idea that empowering women to assert control over reproductive choices is crucial in improving family planning outcomes [54].

The relationship between property ownership and contraceptive utilization suggests a connection between economic empowerment and autonomy in reproductive health choices. As an indicator of socioeconomic status (SES), ownership reflects financial stability and access to material assets that enable individuals to make informed health decisions. This aligns with fundamental cause theory, which posits that individuals with greater resources are better equipped to maintain good health [55]. Similarly, prior evidence also supports the role of physical capital in shaping contraceptive behavior, particularly in resource-limited settings [56].

Several sociodemographic variables showed significant associations with contraceptive utilization. The number of living children plays a key role in contraceptive adoption, as women who have achieved their preferred family size tend to be more motivated to prevent additional births. Consistent with previous studies [21, 24], the number of living children has emerged as a major influence on contraceptive behavior. These findings imply the ongoing importance of fertility preferences in shaping contraceptive use.

Education serves as a fundamental determinant of contraceptive uptake, as it equips women with the knowledge and awareness needed for informed reproductive decision-making. The results of this study align with evidence from sub-Saharan Africa, emphasizing the role of education in facilitating informed contraceptive choices [16, 18, 21, 23, 51]. By enhancing decision-making skills, fostering financial independence, and improving social standing, education enables women to overcome barriers that limit their reproductive autonomy [29]. These findings underscore that educational attainment enhances access to reproductive health resources and enables women to develop the skills and confidence needed to achieve positive health outcomes [26].

Economic circumstances are central in determining access to and utilization of modern contraceptive and family planning services. The findings of the study align with global evidence emphasizing the impact of financial resources on modern contraceptive use [17, 21, 24, 57, 58]. Women with greater economic means have increased access to health care services, the financial ability to afford contraceptives, and the capacity to make informed family planning choices [55].

Employment status is a critical factor influencing modern contraceptive use, with employed women, particularly those earning wages, demonstrating higher contraceptive uptake than non-employed women do. This is consistent with global research showing that workforce participation strengthens women’s economic empowerment, fostering greater autonomy in decision-making, increasing knowledge and awareness, and enhancing access to healthcare services, ultimately resulting in better reproductive health outcomes [23, 59]. Women in high-paying jobs or working in government increasingly prioritize planned pregnancies to balance their careers and family responsibilities, reflecting the substantial opportunity costs associated with childbearing for those in the workforce [60]. When childcare responsibilities restrict women’s involvement in the labor force, access to effective contraception enables them to regulate reproductive timing, underscoring the need to address structural barriers to both employment and reproductive autonomy [61].

Exposure to family planning information significantly enhanced modern contraceptive use, as observed in this study and supported by prior research [21, 23, 62, 63]. Such exposures create an environment where contraceptive practices are normalized and socially accepted, motivating women to adopt family planning methods. Media campaigns provide essential sexual and reproductive health information, enabling informed decision-making [63].

Taken together, these findings suggest the need for targeted and equity-driven policy responses. First, Tanzania’s family planning programs should adopt a more holistic, rights-based approach that positions women’s empowerment at the center of program design and delivery. This includes not only expanding access to contraceptive methods but also supporting women’s autonomy through greater participation in household and sexual decision-making. Programs should actively involve men and boys to promote joint decision-making and foster more gender-equitable norms, while leveraging behavior change strategies—such as community dialogues, media campaigns, and gender-transformative education—to reshape attitudes and reduce stigma [17, 64, 65]. Second, policies should address the structural determinants of reproductive autonomy, such as poverty, unequal property rights, and limited access to education and formal employment. Targeted efforts to reach economically disadvantaged women, adolescents, and rural populations are critical. Finally, continued investment in social and behavior change communication across multiple platforms will be essential to sustaining demand, expanding access, and supporting informed contraceptive choices [62].

### Strengths and limitations

This study has several limitations. First, its cross-sectional design restricts causal inference between women’s empowerment and contraceptive use. For example, women already engaged in family planning may be more likely to participate in household decision-making. While some qualitative studies suggest a temporal link between changes in gender norms and reproductive behaviors [38], longitudinal and experimental studies are needed to confirm these pathways. Second, this study included only currently married or cohabiting women, as DHS empowerment indicators—such as decision-making and sexual negotiation—are available only for this group. As such, the findings are not intended to generalize to unmarried or adolescent women, who may face distinct barriers to contraceptive uptake and contribute to population growth [33, 66]. This exclusion does not reflect a methodological bias, but rather a deliberate analytic focus aligned with the available data. Nonetheless, given Tanzania’s Family Planning 2030 commitment to expand youth-friendly services [41], future studies should explore empowerment and contraceptive use among unmarried and adolescent populations. Third, women’s empowerment is shaped by social, cultural, and economic contexts, and its relationship with modern contraceptive practices also varies across different levels of a national development. This study, which was conducted in the developing context of Tanzania, reflects specific local conditions for understanding the impact of empowerment on reproductive health. These findings suggest the need for extensive comparative research across diverse settings in developing countries to provide more nuanced insight into how empowerment influences women’s reproductive health outcomes.

Despite these limitations, the study offers notable strengths. It draws on the most recent nationally representative data from the 2022 TDHS, ensuring reliability and generalizability within the population of currently married or cohabiting women. The analysis employed a multidimensional empowerment index encompassing five domains and 18 items, capturing a broad range of empowerment indicators relevant to modern contraceptive use. Key empowerment proxies included household decision-making, sexual negotiation, gender-equitable attitudes, property ownership, and social independence. In addition, the study controlled for other important factors such as age at significant life events, fertility, education, wealth, wage employment, and exposure to family planning messages. This multidimensional approach enhances the interpretability and policy relevance of the findings.

## Conclusions

Building on the strengths of a nationally representative dataset and a multidimensional empowerment framework, this study examined how various aspects of women’s empowerment are associated with modern contraceptive use in Tanzania. The findings indicate that greater autonomy in household and sexual decision-making, access to economic resources, and exposure to family planning information are each linked to higher contraceptive uptake among married women.

These results suggest that empowering women across multiple dimensions may support more consistent use of modern contraceptives. However, contraceptive behavior is shaped not only by individual agency but also by broader social and structural conditions. While this study focused on currently married women, the findings underscore the importance of addressing systemic barriers, such as restrictive gender norms, economic disadvantage, and limited access to information, that hinder informed reproductive choices.

To improve contraceptive uptake in Tanzania, future programs should incorporate both individual-level and structural-level strategies that strengthen women’s reproductive agency. By aligning women’s empowerment efforts with broader health and development goals, policymakers can design more context-sensitive and sustainable approaches to advancing reproductive health. These findings add to the growing body of evidence on the role of women’s empowerment in influencing contraceptive behavior and may inform evidence-based, context-specific strategies in similar low-resource settings.

## Data Availability

The datasets used in the analysis are freely available from the Demographic and Health Survey at https://dhsprogram.com/Countries/Country-Main.cfm? ctry_id=39.

## Abbreviations

AORs: Adjusted Odds Ratios
CIs: Confidence Intervals
IUDs: Intrauterine Devices
LAM: Lactational Amenorrhea Method
mCPR: Modern Contraceptive Prevalence Rate
SDGs: Sustainable Development Goals
SDM: Standard Days Method
SES: Socioeconomic Status
SWPER: Survey-based Women’s Empowerment Index
TDHS: Tanzania Demographic and Health Survey
TFR: Total Fertility Rate
USAID: United States Agency for International Development
VIFs: Variance Inflation Factors

## References

[1] Ministry of Finance National Bureau of Statistics Tanzania, Presidents’ Office, Ministry of Finance and Planning Office of the Chief Government Statistician, Zanzibar. BASIC DEMOGRAPHIC AND SOCIO-ECONOMIC PROFILE Key Findings [Internet]. 2024. Available from: https://sensa.nbs.go.tz/publication/08.%20Key_Findings_Basic_Demographic_and_Socio-economic_%20Eng_%2012.06.2024%20Final.pdf. Accessed 18 Apr 2025.

[2] United Nations, Department of Economic and Social Affairs, Population Division. World Population Prospects 2022, online edition. https://population.un.org/wpp/. Accessed 18 Apr 2025.

[3] Migrants and refugees section. Country profile: United Republic of Tanzania. Vatican City: dicastery for promoting integral human development. 2020. https://migrants-refugees.va/country-profile/tanzania. Accessed 18 Apr 2025.

[4] Agwanda A. Population growth, structure and momentum in Tanzania. Nairobi: University of Nairobi; 2014.

[5] Loiboo D, Luvanda E, Osoro N. Population and economic growth in Tanzania. Tanzan J Popul Stud Dev. 2021;28(2):20–42. 10.56279/tjpsd.v28i2.125.

[6] Ministry of Health (MoH). [Dodoma], Ministry of Health (MoH) [Zanzibar], National Bureau of Statistics (NBS), Office of the Chief Government Statistician (OCGS), and ICF. Tanzania Demographic and Health Survey and Malaria Indicator Survey (TDHS-MIS) 2022. Dodoma, Rockville: MoH, NBS, OCGS, and ICF; 2023. https://dhsprogram.com/pubs/pdf/FR382/FR382.pdf.

[7] Bongaarts J. Trends in fertility and fertility preferences in sub-Saharan Africa: the roles of education and family planning programs. Genus. 2020;76(1):32. 10.1186/s41118-020-00098-z.

[8] Bongaarts J. A framework for analyzing the proximate determinants of fertility. Popul Dev Rev. 1978;4(1):105–32. 10.2307/1972149.

[9] Cleland J, Conde-Agudelo A, Peterson H, Ross J, Tsui A. Contraception and health. Lancet. 2012;380(9837):149–56. 10.1016/S0140-6736(12)60609-6. 22784533 10.1016/S0140-6736(12)60609-6

[10] Kenya National Bureau of Statistics (KNBS) and ICF. Kenya Demographic and Health Survey 2022: Key Indicators Report. Nairobi, Kenya and Rockville, Maryland, USA: KNBS and ICF; 2023. https://dhsprogram.com/pubs/pdf/PR143/PR143.pdf.

[11] National Institute of Statistics of Rwanda (NISR). Ministry of health (Rwanda), and ICF. Rwanda demographic and health survey 2019–20. Kigali. Rockville: NISR, MoH, and ICF; 2021.

[12] Track20 Project. Malawi: Family Planning Opportunity Brief. https://www.track20.org/download/pdf/Opportunity%20Briefs/english/Malawi%20FP%20Opportunity%20Brief.pdf. Accessed 18 Apr 2025.

[13] Boadu I. Coverage and determinants of modern contraceptive use in sub-Saharan Africa: further analysis of demographic and health surveys. Reprod Health. 2022;19(1):18. 10.1186/s12978-022-01332-x. 35062968 10.1186/s12978-022-01332-x PMC8781110

[14] Upadhyay UD, Gipson JD, Withers M, Lewis S, Ciaraldi EJ, Fraser A, et al. Women’s empowerment and fertility: a review of the literature. Soc Sci Med. 2014;115:111–20. 10.1016/j.socscimed.2014.06.014. 24955875 10.1016/j.socscimed.2014.06.014 PMC4096045

[15] Prata N, Fraser A, Huchko MJ, Gipson JD, Withers M, Lewis S, et al. Women’s empowerment and family planning: a review of the literature. J Biosoc Sci. 2017;49(6):713–43. 10.1017/S0021932016000663. 28069078 10.1017/S0021932016000663 PMC5503800

[16] Asaolu IO, Okafor CT, Ehiri JC, Dreifuss HM, Ehiri JE. Association between measures of women’s empowerment and use of modern contraceptives: an analysis of Nigeria’s demographic and health surveys. Front Public Health. 2017;4:293. 10.3389/fpubh.2016.00293. 28119909 10.3389/fpubh.2016.00293 PMC5220063

[17] Mathur S, Kirk K, Dadi C, Dougherty L. Women’s involvement in decision-making and association with reproductive health behaviors: findings from a cross-sectional survey in Niger. BMC Womens Health. 2024;24(1):278. 10.1186/s12905-024-03115-x. 38715013 10.1186/s12905-024-03115-x PMC11075281

[18] Woya AA, Tekile AK, Mitiku AA. Women’s empowerment on contraceptive use among married women in Amhara Region, Ethiopia. ISABB J Health Environ Sci. 2018;5(1):1–8.

[19] Bhowmik J, Biswas RK, Williams J, Dey SR. Women’s decision-making power can influence modern contraceptive use: evidence from Bangladesh. Int J Health Plann Manage. 2024;39(5):1503–15. 10.1002/hpm.3822. 39032057 10.1002/hpm.3822

[20] Jamali Y, Simon DJ. Modern contraception in Pakistan: a cross-sectional study. Popul Econ. 2024;8(1):77–96. 10.3897/popecon.8.e106872.

[21] Kraft JM, Serbanescu F, Schmitz MM, Mwanshemele Y, Ruiz CAG, Maro G, et al. Factors associated with contraceptive use in sub-Saharan Africa. J Womens Health (Larchmt). 2022;31(3):447–57. 10.1089/jwh.2020.8984. 34129385 10.1089/jwh.2020.8984 PMC8972023

[22] Kidayi PL, Msuya S, Todd J, Mtuya CC, Mtuy T, Mahande MJ. Determinants of modern contraceptive use among women of reproductive age in tanzania: evidence from Tanzania demographic and health survey data. Adv Sex Med. 2015;5(3):43–52. 10.4236/asm.2015.53006.

[23] Yaya S, Uthman OA, Ekholuenetale M, Bishwajit G. Women empowerment as an enabling factor of contraceptive use in sub-Saharan Africa: a multilevel analysis of cross-sectional surveys of 32 countries. Reprod Health. 2018;15(1):214. 10.1186/s12978-018-0658-5. 30572927 10.1186/s12978-018-0658-5 PMC6302468

[24] Demeke H, Legese N, Nigussie S. Modern contraceptive utilization and its associated factors in East Africa: findings from multi-country demographic and health surveys. PLoS One. 2024;19(1):e0297018. 10.1371/journal.pone.0297018. 38241359 10.1371/journal.pone.0297018 PMC10798634

[25] Kabeer N. The conditions and consequences of choice: reflections on the measurement of women’s empowerment. Geneva: UNRISD; 1999.

[26] Kabeer N. Resources, agency, achievements: reflections on the measurement of women’s empowerment. Dev Change. 1999;30(3):435–64. 10.1111/1467-7660.00125.

[27] Narayan-Parker D. Empowerment and poverty reduction: a sourcebook. Washington, DC: World Bank; 2002.

[28] Pradhan B. Measuring empowerment: a methodological approach. Development. 2003;46(2):51–7. 10.1057/palgrave.development.1110445.

[29] Kabeer N. Gender equality and women’s empowerment: a critical analysis of the third millennium development goal 1. Gend Dev. 2005;13(1):13–24. 10.1080/13552070512331332273.

[30] Ewerling F, Lynch JW, Victora CG, van Eerdewijk A, Tyszler M, Barros AJD. The SWPER index for women’s empowerment in africa: development and validation of an index based on survey data. Lancet Glob Health. 2017;5(9):e916–23. 10.1016/S2214-109X(17)30292-9.​. 28755895 10.1016/S2214-109X(17)30292-9 PMC5554795

[31] Holt K, Challa S, Alitubeera P, Atuyambe L, Dehlendorf C, Galavotti C, et al. Conceptualizing contraceptive agency: a critical step to enable human rights-based family planning programs and measurement. Glob Health Sci Pract. 2024;12(1):e2300299. 10.9745/GHSP-D-23-00299. 38346841 10.9745/GHSP-D-23-00299 PMC10906552

[32] Bowers SF, Lambert VJ, Nzali A, Samson A, Mwakisole N, Yahaya H, et al. Proposed changes to framework to assess contraceptive autonomy based on phased in-depth interviews in Northwest Tanzania. Reprod Health. 2025;22(1):24. 10.1186/s12978-025-01963-w. 39955598 10.1186/s12978-025-01963-w PMC11829388

[33] Matovu JKB, Amongin D, Akulume M, Tuhebwe D, Murungi I, Wanyenze RK. Contraception decision-making autonomy among adolescent girls and young women in Uganda. NPJ Womens Health. 2025;3(1):14. 10.1038/s44294-025-00066-y.

[34] Ewerling F, Raj A, Victora CG, Hellwig F, Coll CV, Barros AJ. Swper global: a survey-based women’s empowerment index expanded from Africa to all low- and middle-income countries. J Glob Health. 2020;10(2):020343. 10.7189/jogh.10.020434. 10.7189/jogh.10.020434 PMC7699005 33274055

[35] Mganga AE, Renju J, Todd J, Mahande MJ, Vyas S. Development of a women’s empowerment index for Tanzania from the demographic and health surveys of 2004–05, 2010, and 2015–16. Emerg Themes Epidemiol. 2021;18(1):13. 10.1186/s12982-021-00094-5. 34620177 10.1186/s12982-021-00103-6 PMC8499508

[36] Mandal M, Muralidharan A, Pappa S. A review of measures of women’s empowerment and related gender constructs in family planning and maternal health program evaluations in low- and middle-income countries. BMC Pregnancy Childbirth. 2017;17(Suppl 2):342. 10.1186/s12884-017-1500-8. 29143636 10.1186/s12884-017-1500-8 PMC5688455

[37] Nanda G, Schuler SR, Lenzi R. The influence of gender attitudes on contraceptive use in tanzania: new evidence using husbands’ and wives’ survey data. J Biosoc Sci. 2013;45(3):331–44. 10.1017/S0021932012000715. 23312349 10.1017/S0021932012000855 PMC3607277

[38] Schuler SR, Rottach E, Mukiri P. Gender norms and family planning decision-making in Tanzania: a qualitative study. J Public Health Afr. 2011;2(2):e25. 10.4081/jphia.2011.e25. 28299066 10.4081/jphia.2011.e25 PMC5345498

[39] Mwalupani N, Sagumo M, Msese N. Determinants of informed decision-making on sexual relations, contraceptive use, and reproductive health among women of reproductive age: a case of married women in Tanzania. East Afr J Arts Soc Sci. 2024;7(1):484–94. 10.37284/eajass.7.1.2088.

[40] Yussuf MH, Elewonibi BR, Rwabilimbo MM, Mboya IB, Mahande MJ. Trends and predictors of changes in modern contraceptive use among women aged 15–49 years in Tanzania from 2004–2016: evidence from Tanzania demographic and health surveys. PLoS One. 2020;15(6):e0234980. 10.1371/journal.pone.0234980. 32598371 10.1371/journal.pone.0234980 PMC7323946

[41] FP2030 Tanzania government commitment [Internet]. Available from: https://www.fp2030.org/tanzania/. Accessed 18 Apr 2025.

[42] United Nations Development Programme (UNDP). Progress on the Sustainable Development Goals: Gender Snapshot 2019 [Internet]. 2019. https://www.undp.org/armenia/publications/progress-sustainable-development-goals-gender-snapshot-2019.

[43] United Nations, Department of Economic and Social Affairs, Population Division. Family Planning and the 2030 Agenda for Sustainable Development: Data Booklet 2019 [Internet]. 2019. https://www.un.org/en/development/desa/population/publications/pdf/family/familyPlanning_DataBooklet_2019.pdf.

[44] Hubacher D, Trussell J. A definition of modern contraceptive methods. Contraception. 2015;92(5):420–1. 10.1016/j.contraception.2015.08.008. 26276245 10.1016/j.contraception.2015.08.008

[45] Rutstein SO, Johnson K. The DHS Wealth Index. DHS Comparative Reports No. 6 [Internet]. Calverton, Maryland: ORC Macro. 2004. https://dhsprogram.com/pubs/pdf/cr6/cr6.pdf.

[46] Asaolu IO, Alaofè H, Gunn JKL, Adu AK, Monroy AJ, Ehiri JE, et al. Measuring women’s empowerment in sub-Saharan Africa: exploratory and confirmatory factor analyses of the demographic and health surveys. Front Psychol. 2018;9:994. 10.3389/fpsyg.2018.00994. 29971030 10.3389/fpsyg.2018.00994 PMC6018402

[47] Bamusi MT, Philip NE, Bhat LD. Women’s empowerment and its influence on the uptake of breast cancer screening in Tanzania: an analysis of 2022 Tanzania demographic health survey data. BMC Womens Health. 2024;24(1):495. 10.1186/s12905-024-03345-z. 39243087 10.1186/s12905-024-03345-z PMC11378501

[48] Ethiopian Public Health Institute (EPHI). Federal ministry of health (FMoH), and ICF. Ethiopia demographic and health survey 2019. Maryland, USA: EPHI and ICF;: Addis Ababa, Ethiopia and Rockville; 2021.

[49] Uganda Bureau of Statistics (UBOS). Ministry of health (MoH) [Uganda], and ICF. Uganda demographic and health survey 2022. Maryland, USA: UBOS and ICF;: Kampala, Uganda and Rockville; 2023.

[50] National Population Commission (NPC). [Nigeria] and ICF. Nigeria demographic and health survey 2018. Abuja, Nigeria and Rockville. Maryland, USA: NPC and ICF; 2019.

[51] Samari G. Women’s empowerment and short- and long-acting contraceptive method use in Egypt. Cult Health Sex. 2017;20(4):458–73. 10.1080/13691058.2017.1343270. 28786755 10.1080/13691058.2017.1356938 PMC6103444

[52] Do M, Fu H. Is women’s self-efficacy in negotiating sexual decisionmaking associated with condom use in marital relationships in Vietnam? Stud Fam Plann. 2011;42(4):273–82. 10.1111/j.1728-4465.2011.00290.x. 22292246 10.1111/j.1728-4465.2011.00290.x

[53] Crissman HP, Adanu RM, Harlow SD. Women’s sexual empowerment and contraceptive use in Ghana. Stud Fam Plann. 2012;43(3):201–12. 10.1111/j.1728-4465.2012.00318.x. 23185863 10.1111/j.1728-4465.2012.00318.x

[54] Exavery A, Kanté AM, Jackson E, Noronha J, Sikustahili G, Tani K, et al. Role of condom negotiation on condom use among women of reproductive age in three districts in Tanzania. BMC Public Health. 2012;12:1097. 10.1186/1471-2458-12-1097. 23256530 10.1186/1471-2458-12-1097 PMC3585459

[55] Link BG, Phelan JC. Understanding sociodemographic differences in health—the role of fundamental social causes. Am J Public Health. 1996;86(4):471–3. 10.2105/AJPH.86.4.471. 8604773 10.2105/ajph.86.4.471 PMC1380543

[56] González C, Houweling TAJ, Marmot MG, Brunner EJ. Comparison of physical, public and human assets as determinants of socioeconomic inequalities in contraceptive use in Colombia—moving beyond the household wealth index. Int J Equity Health. 2010;9:10. 10.1186/1475-9276-9-10. 20380713 10.1186/1475-9276-9-10 PMC2873465

[57] Adebowale SA, Adedini SA, Ibisomi LD, Palamuleni ME. Differential effect of wealth quintile on modern contraceptive use and fertility: evidence from Malawian women. BMC Womens Health. 2014;14:40. 10.1186/1472-6874-14-40. 24602452 10.1186/1472-6874-14-40 PMC3973841

[58] Tampah-Naah AM, Yendaw E, Sumankuuro J. Residential status and household wealth disparities in modern contraceptives use among women in Ghana: a cross-sectional analysis. BMC Womens Health. 2023;23(1):550. 10.1186/s12905-023-02684-7. 37875940 10.1186/s12905-023-02684-7 PMC10594689

[59] Shapiro D, Tambashe BO. The impact of women’s employment and education on contraceptive use and abortion in Kinshasa, Zaire. Stud Fam Plann. 1994;25(2):96–110. 10.2307/2138087. 8059449

[60] John NA, Tsui AO, Roro M. Quality of contraceptive use and women’s paid work and earnings in peri-urban Ethiopia. Fem Econ. 2020;26(1):23–43. 10.1080/13545701.2019.1632471.

[61] Pekkurnaz D. Employment status and contraceptive choices of women with young children in Turkey. Fem Econ. 2020;26(1):98–120. 10.1080/13545701.2019.1642505.

[62] Sserwanja Q, Turimumahoro P, Nuwabaine L, Kamara K, Musaba MW. Association between exposure to family planning messages on different mass media channels and the utilization of modern contraceptives among young women in Sierra leone: insights from the 2019 Sierra Leone demographic health survey. BMC Womens Health. 2022;22(1):376. 10.1186/s12905-022-02012-1. 36114503 10.1186/s12905-022-01974-w PMC9479264

[63] Ghosh R, Mozumdar A, Chattopadhyay A, Acharya R. Mass media exposure and use of reversible modern contraceptives among married women in India: an analysis of the NFHS 2015–16 data. PLoS One. 2021;16(7):e0254400. 10.1371/journal.pone.0254400. 34255787 10.1371/journal.pone.0254400 PMC8277022

[64] Skinner J, Hempstone H, Raney L, Galavotti C, Light B, Weinberger M, et al. Elevating social and behavior change as an essential component of family planning programs. Stud Fam Plann. 2021;52(3):383–93. 10.1111/sifp.12169. 34268743 10.1111/sifp.12169 PMC8457161

[65] Shattuck D, Kerner B, Gilles K, Hartmann M, Ng’ombe T, Guest G. Encouraging contraceptive uptake by motivating men to communicate about family planning: the Malawi male motivator project. Am J Public Health. 2011;101(6):1089–95. 10.2105/AJPH.2010.300091. 21493931 10.2105/AJPH.2010.300091 PMC3093271

[66] Kagoye SA, Jahanpour O, Ngoda OA, Obure J, Mahande MJ, Renju J. Trends and determinants of unmet need for modern contraception among adolescent girls and young women in Tanzania, 2004–2016. PLOS Glob Public Health. 2024;4(1):e0000695. 10.1371/journal.pgph.0000695. 38170707 10.1371/journal.pgph.0000695 PMC10763936

